# Supplementary material for: A SARS-CoV-2 variant elicits an antibody response with a shifted immunodominance hierarchy
Source: PLoS Pathog. 2022 Feb 8;18(2):e1010248. doi: 10.1371/journal.ppat.1010248 (PMC8856557; doi:10.1371/journal.ppat.1010248)
Supplement: Attachment — Submitted filename: 211218_reviewer_response.pdf [file ppat.1010248.s011.pdf]

We thank the Reviewers for the careful and helpful review of the manuscript. We believe the reviews were careful and fair, and that the suggested edits to the manuscript improve its clarity. We have addressed all the Reviewers' concerns (see below for full details). The Reviewer comments are in black, and our responses are in blue.

Reviewer Comments (if any, and for reference):

Reviewer's Responses to Questions
Part I - Summary
Please use this section to discuss strengths/weaknesses of study, novelty/significance, general execution and scholarship.

Reviewer #1: Greaney et al. describe experiments comparing the binding and neutralizing antibody specificities in convalescent plasma from 9 persons infected with B.1.351 SARS-CoV-2 variants in the Republic of South Africa to convalescent plasma from 17 persons infected with early 2020 SARS-CoV-2 variants in the US. In lentiviral pseudovirus neutralization assays using ACE2-293T target cells, the authors show that depleting plasma of anti-RBD antibodies removes most of the neutralization activity in the B.1.351 convalescent plasma, as was seen previously for early 2020 variant convalescent plasma. Using the deep mutational scanning method to evaluate the effects of most possible single mutations in the RBD on antibody binding in B.1.351 plasma, they further show that 4/9 samples were predominantly affected by 484 mutation but 3/9 samples were also significantly impacted by 443-450, 498-501, as well as 484 mutations. This contrasted findings from 11 early 2020 plasma samples that were predominantly impacted by 484, 486, and 456 mutations. Neutralization experiments also showed that early 2020 convalescent plasma was greatly affected by E484K/Q and K417N-E484K-N501Y mutations while the impacts of these mutations were more modest for B.1.351 convalescent plasma. B.1.351 plasma samples also tended to be more affected by the G446V mutation. The authors conclude that SARS-CoV-2 variants can elicit polyclonal antibodies with different immunodominance hierarchies.

Experimental methods are strong, innovative, and carefully documented, with no major weaknesses. Conclusions are supported by the data. The authors acknowledge the major limitations of their studies, including use of a neutralization assay that detect mostly RBD-directed antibodies, RBD binding assays that do not completely mimic the trimeric Spike on virions, and the relatively small number of samples. The findings offer a valuable extension of prior studies by providing more detailed characterization of differences in antibody specificities elicited by B.1.351 SARS-CoV-2 infections compared to early 2020 SARS-CoV-2

variant infections. These results have implications for our understanding of immunity elicited by different SARS-CoV-2 variants.

We thank the Reviewer for their positive comments and appreciation of the study.

Reviewer #2: Greaney et al utilized a previously established, high throughput and unbiased mutagenesis system to study plasma antibody interactions with neutralizing SARS-CoV-2 viral epitopes located on the receptor binding domain of the Spike protein. They have previously applied this method with great success to an early strain of SARS-CoV-2. Here, they apply the same system to convalescent plasma from individuals infected with the beta variant, B.1.351, and identify differential antibody binding preferences and neutralizing specificities. While this reviewer is enthusiastic about the presentation and analysis of their data sets, some of the caveats of the study are not sufficiently emphasized. Overall, however, the study provides important data on serum antibody recognition of the beta variant.

We thank the Reviewer for their comments and appreciation of the study and are happy to address their concerns below.

Part II – Major Issues: Key Experiments Required for Acceptance
Please use this section to detail the key new experiments or modifications of existing experiments that should be absolutely required to validate study conclusions.

Generally, there should be no more than 3 such required experiments or major modifications for a "Major Revision" recommendation. If more than 3 experiments are necessary to validate the study conclusions, then you are encouraged to recommend "Reject".

Reviewer #1: (No Response)
Reviewer #2: none

Part III – Minor Issues: Editorial and Data Presentation Modifications
Please use this section for editorial suggestions as well as relatively minor modifications of existing data that would enhance clarity.

Reviewer #1: (No Response)
Reviewer #2: Please comment in the discussion about how the arbitrary threshold of 3-6% gating might bias the results, particularly in the context of comparison of their new data sets with their old data sets. I note there is a mix of linear and curved gates, with the linear gating strongly favoring variants with low RBD expression. Some of the curved gates do as well.

The goal is to identify the 5% of RBD-expressing yeast cells with the lowest amount of antibody binding for their degree of RBD expression. We set the antibody-escape gates to try to capture the lowest 5% of each replicate library, but it can be challenging to get exactly 5%, given that small changes in the shape of the gate can lead to relatively large shifts in the percent of cells that fall into the gate. We agree with the reviewer that for some samples variants with lower RBD expression are favored in the FACS scheme. We set the curved gates for samples for which there is not a clear ~5% population with substantially reduced antibody binding. We hypothesized that these might be samples with relatively more broad binding to the RBD than others, and that by setting a curved ~5% gate, we might expect to find a broad antibody-escape profile, with no mutations leading to disproportionate reductions in binding. To our modest surprise, samples with curved gates (K046, K114, K119) did not seem to have flat escape profiles. Rather, they had the 484+class 3 phenotype. In general, variants containing mutations with strongly deleterious effects on RBD expression do fall into the antibody-escape gate, but these are computationally filtered out as "spurious" mutations based on an RBD expression filter of -1.0 and an ACE2-binding filter of -3.0, as determined by high-throughput measurements of the effects of these mutations using these same B.1.351 RBD mutant libraries on RBD expression and ACE2 binding.

We have added the following text to the main text Results section describing the approach: "FACS selection gates are set to capture the approximately 5% of cells with the lowest amount of antibody binding for their amount of RBD expression. This involves some subjectivity, which may affect which mutations are identified as antibody-escape variants."

Please briefly mention why 484 -> V, F, P, L etc weren't included in the pseudotyped virus neutralization analysis, since those mutations appeared to cause the most consistent reduction in antibody binding to RBD based on the mutational scanning. Correspondingly, the authors mention "but within the RBD, site 484 is less immunodominant for B.1.351-elicited plasmas" - presumably this refers only to the effect on neutralization. If so, this claim may need to be revised or substantiated by measurements of neutralization against pseudotyped viruses expressing V, F, P, or L mutations.

We focus our neutralization assays on mutations that are most prevalent among circulating viral isolates, as these are the results that are likely to be most relevant when considering the effects of mutations in viral variants on antibody immunity. At the time of the study (Aug. 1, 2021) there were >3 million spike sequences deposited in GISIAD. At site 484, only the E484K and E484Q mutations were present at a frequency above 0.1%. The E484A mutation had also been observed at a frequency of 0.00377%. In the past few weeks of course, the E484A mutation has

gained more notoriety for being present in the Omicron (B.1.1.529) variant. The V mutation had been observed at a frequency of 4.34e-6, and may be due to sequencing or alignment errors. The F, P, and L mutations had not been observed in any sequenced isolates. We agree with the reviewer and moderate our statements. Accordingly we have revised the text to say, "but within the RBD, the E484K and E484Q mutations, which have large effects on early 2020 plasmas, have more moderate effects on neutralization by B.1.351-elicited plasmas."

Why is L452R excluded from fig 5? The authors show the NT data in the supplement with all of the others, but it isn't in the main body.

We found a relatively small effect of the L452R on neutralization by B.1.351-elicited plasmas, so we did not test the effect of this mutation on the corresponding early 2020 plasmas, as there is already an abundance of literature describing this effect. We therefore did not include L452R in Figure 5 since that figure explicitly compares mutations tested on both plasma sets. However, we still wanted to include the data in the supplement even if it didn't make sense to include in the comparative main figure.

No tested mutation, nor the 417-484-501 triple mutant, reduced neutralization by the B.1.351 plasmas as much as removing all RBD-binding antibodies (Fig. 5) – could the authors expand on the significance of this finding in the discussion? Does this have to do with elevated RBD polyclonality in the B.1.351 plasmas? Would this be a virus effect or could it be host response effect?

We agree that it is interesting that no tested mutation, nor the 417-484-501 triple mutant, reduced neutralization by the B.1.351 plasmas as much as removing all RBD-binding antibodies. We speculate in the Discussion about possible virus and host factors (e.g., germline gene utilization). We also add the following text, "Specifically, the early 2020 samples were collected in early 2020 in Washington State, USA, and the B.1.351 samples were collected in December 2020–January 2021 in South Africa. Nevertheless, the two cohorts are relatively well-matched with respect to age, sex, and days-post symptom onset of sample collection (**Table 1**) and assays were performed under comparable conditions. But host factors, including antibody germline gene alleles, immune history, and prior exposures to endemic coronaviruses may contribute to the differences observed in the specificity of the SAR-CoV-2 antibody response."

The claim that there are significant differences in the RBD epitope hierarchies between the B.1.351 and 2020 sera should be tempered or made more specific. There are a couple of reasons for this. First, comparing the escape maps reveals that some responses are actually more similar than different, eg K007/K031/K040 vs participant C and K046/K114/K119 vs participants G and

H. There are thus heterogenous subgroups, so definitive differences would need a larger cohort study to overcome potential sampling artifacts. Another extremely important caveat, which is not sufficiently emphasized, are the likely differences in host factors. These factors include ancestry (and thus HLA genetics, which certainly can influence epitope selection), immune response history (including due to differences in microbiota), and prior exposures to endemic coronaviruses (and thus original antigenic sin).

We thank the reviewer for these insightful comments, with which we agree. We now specifically call out participants C, G, and H from the early 2020 cohort in our discussion of similarities and differences between the two cohorts. We also comment on the small sample sizes of our cohorts. We state, "There is also heterogeneity among the antibody-escape maps within each of the two cohorts as well as similarities between cohorts. For instance, the antibody-escape map for participant C of the early 2020 cohort qualitatively resembles that of the "484-focused" B.1.351 cohort samples, and the maps for participants G and H qualitatively resemble the "484 and class 3-focused" group. Thus, the trends observed here must be interpreted with the caveat that the two cohort sizes are relatively small."

We completely agree that host factors are an important determinant of the specificity of the antibody response. We add text to the Discussion to expand upon this possibility (see response to previous comment above for the full addition).

Figure 3 is not called out in the main text.
Figure 3 is called out in the Results section titled, "B.1.351-elicited antibodies focus on different epitopes than early 2020 convalescent samples," and we have added a callout to this figure in the section, "Complete mapping of mutations in the B.1.351 RBD that reduce binding by polyclonal plasma antibodies elicited by B.1.351 infection".

Minor suggestions:
• Would be nice to have 1-2 sentences in the main text about how the escape system works (yeast display, etc) and how the library was generated.
We have elaborated on how the system works in the main text Results section titled, "Complete mapping of mutations in the B.1.351 RBD that reduce binding by polyclonal plasma antibodies elicited by B.1.351 infection."

• Comment on the advantages/challenges of combinatorial sublibraries (all variants in the context of an 484 mutant for example).
We thank the reviewer for mentioning this, and we agree that this would be a worthwhile study! We are currently working to create RBD deep mutational scanning libraries in other

backgrounds, although it is important to choose the right library and the right sera to test against that library.

We think combinatorial libraries would also be very interesting! Unfortunately, it is not possible to make a library with even all possible combinations of double mutants due to the massive number of possible combinations (~14 million). We are very interested in combinatorial libraries, but due to the challenges of selecting the correct mutations and correct plasma samples to use, it will be a while before such a study is ready.

Unfortunately, we are constrained by how many libraries we can build and how many experiments we can run with available plasma sample volumes. The DMS libraries are relatively expensive to build (~$10k in library synthesis costs), the experiments are relatively expensive to run (~$10–15k in NGS costs) and require several months of experiments and many hours on the FACS machine. The sera/plasma samples are in limited supply, and the experiments require relatively large volumes of plasmas to run. We are trying to pick our next libraries judiciously to spend our efforts on the most high-yield experiments! But we do wish that we could make many more libraries in different and combinatorial backgrounds and compare results across them.

• How were 11 of the 17 2020 samples selected for escape mapping?
The 11 samples were chosen from an initial panel of 17 participants to cover a range of serum binding and neutralizing potencies and degrees of RBD-directed neutralization potencies (see Greaney, et al. (2021), PMID 33592168 for more details). We now add this explicitly to the main text.

• Note in main text that escape mapping involved combined detection of IgG/A/M.
We have added this (see above).

• Please provide the formula cited in reference 37 (so readers don't have to look it up).
We have added this.
Specifically:

$$E_v = F \times \left(n_v^{post} / N_{post}\right) \div \left(n_v^{pre} / N_{pre}\right)$$

where F is the total fraction of the library that escapes antibody binding (these fractions are given as percentages in Fig. S3C), n_v^{post} and n_v^{pre} are the counts of variant v in the RBD library after and before

enriching for antibody-escape variants with a pseudocount of 0.5 added to all counts, and $N_{post} = \sum_v n_v^{post}$ and $N_{pre} = \sum_v n_v^{pre}$ are the total counts of all variants after and before the antibody-escape enrichment. These escape fractions represent the estimated fraction of cells expressing that specific variant that falls in the escape bin, such that a value of 0 means the variant is always bound by plasma and a value of 1 means that it always escapes plasma binding.

- Can the same epitope class coloring be used below the escape fraction plots? Currently just pink and too small.

We have added this to Fig. 3.

- Text in Fig S5 is illegible.

In the final submission, we will ensure that a higher-resolution PDF version of Fig. S5 is included. Currently the journal submission system is limiting the resolution of the file we can upload.
